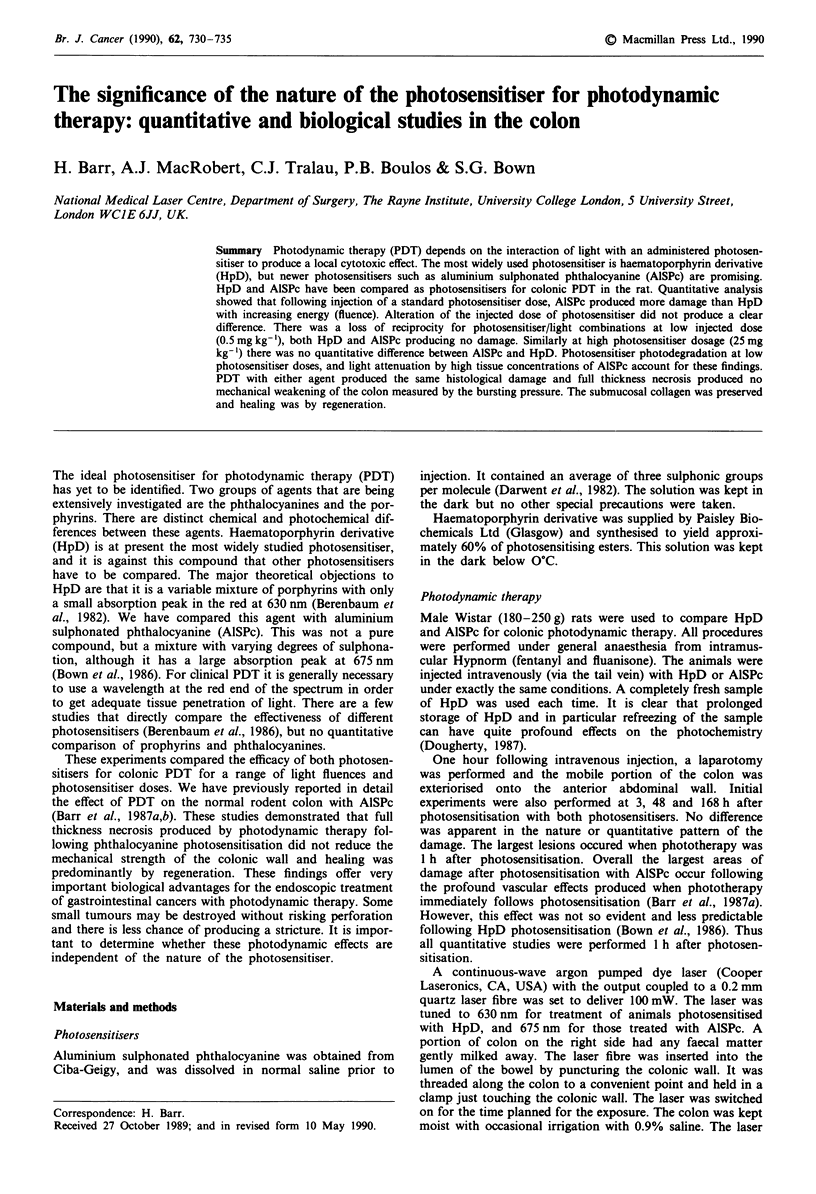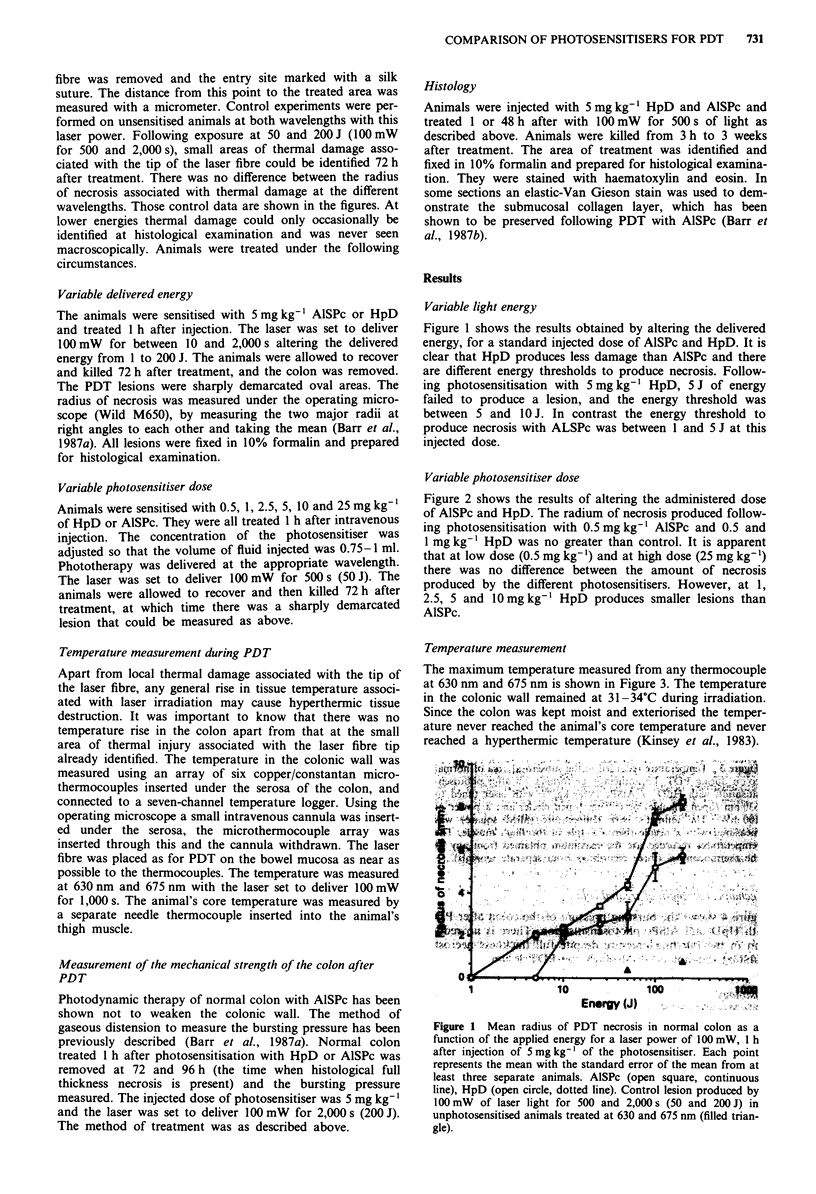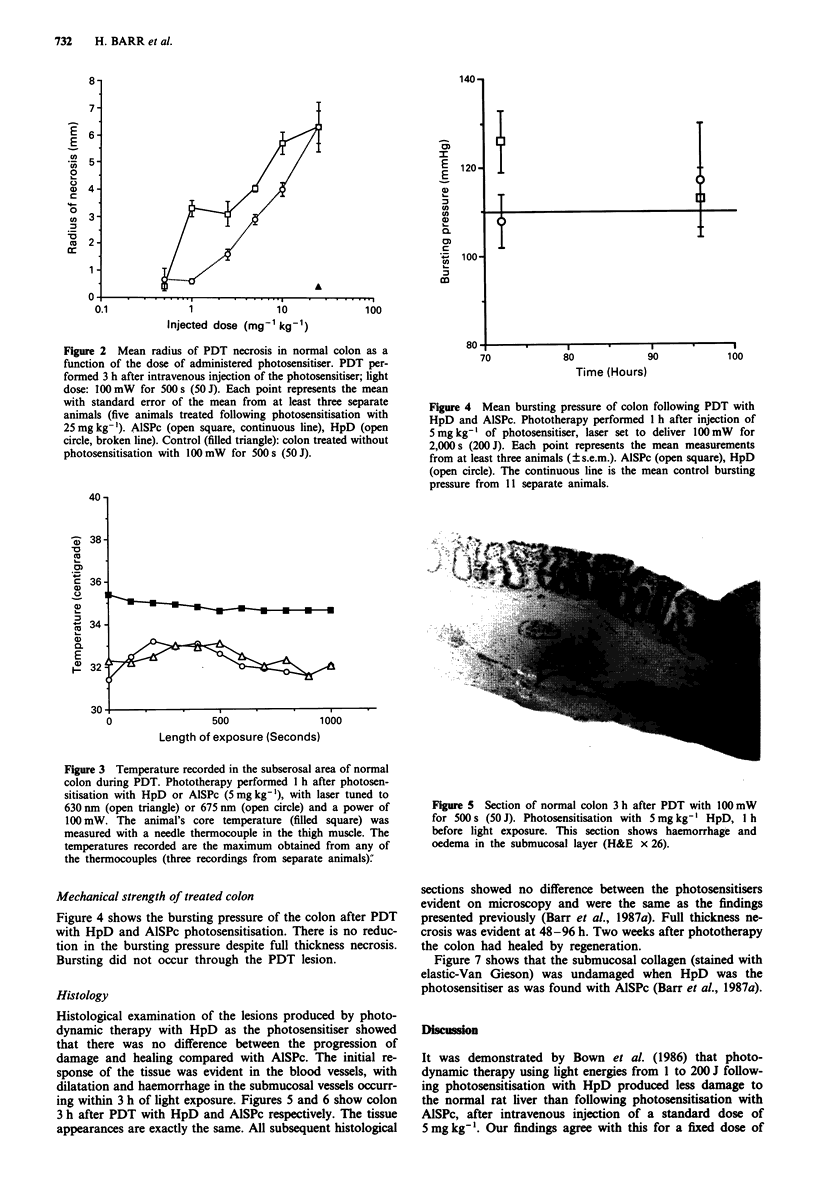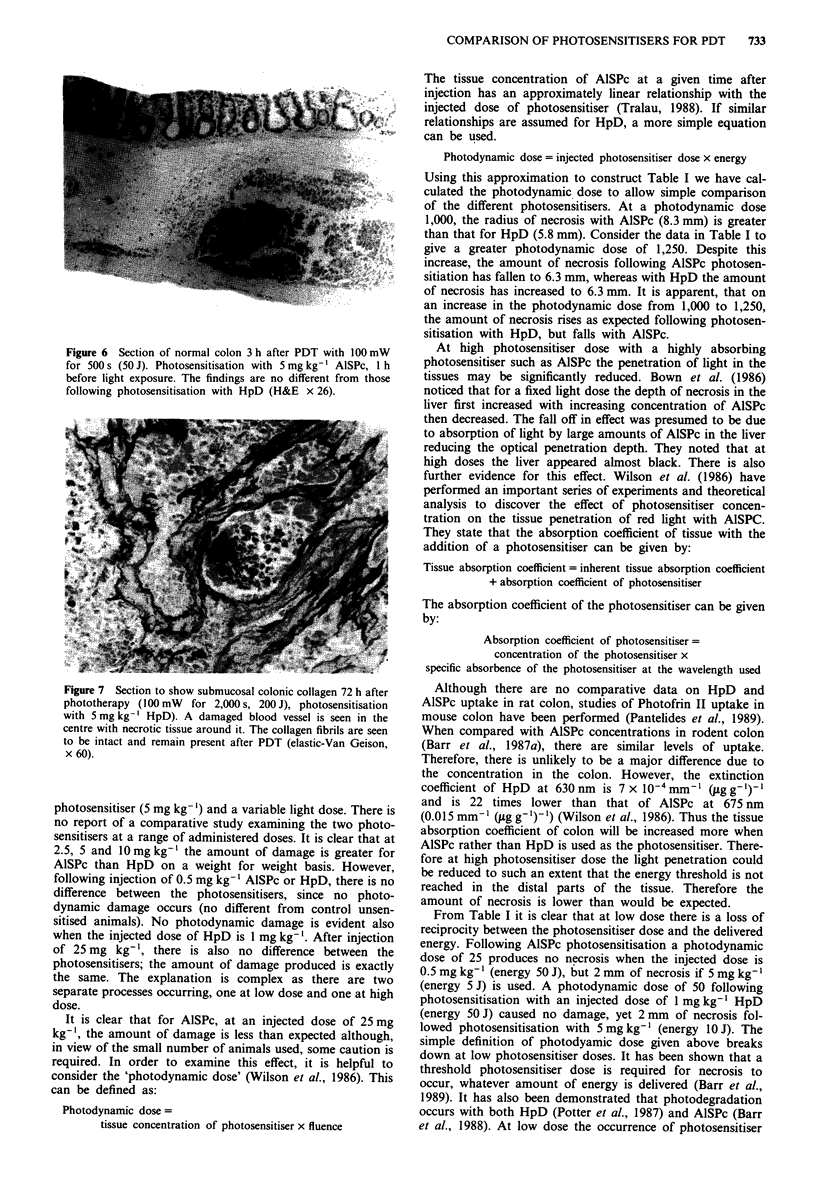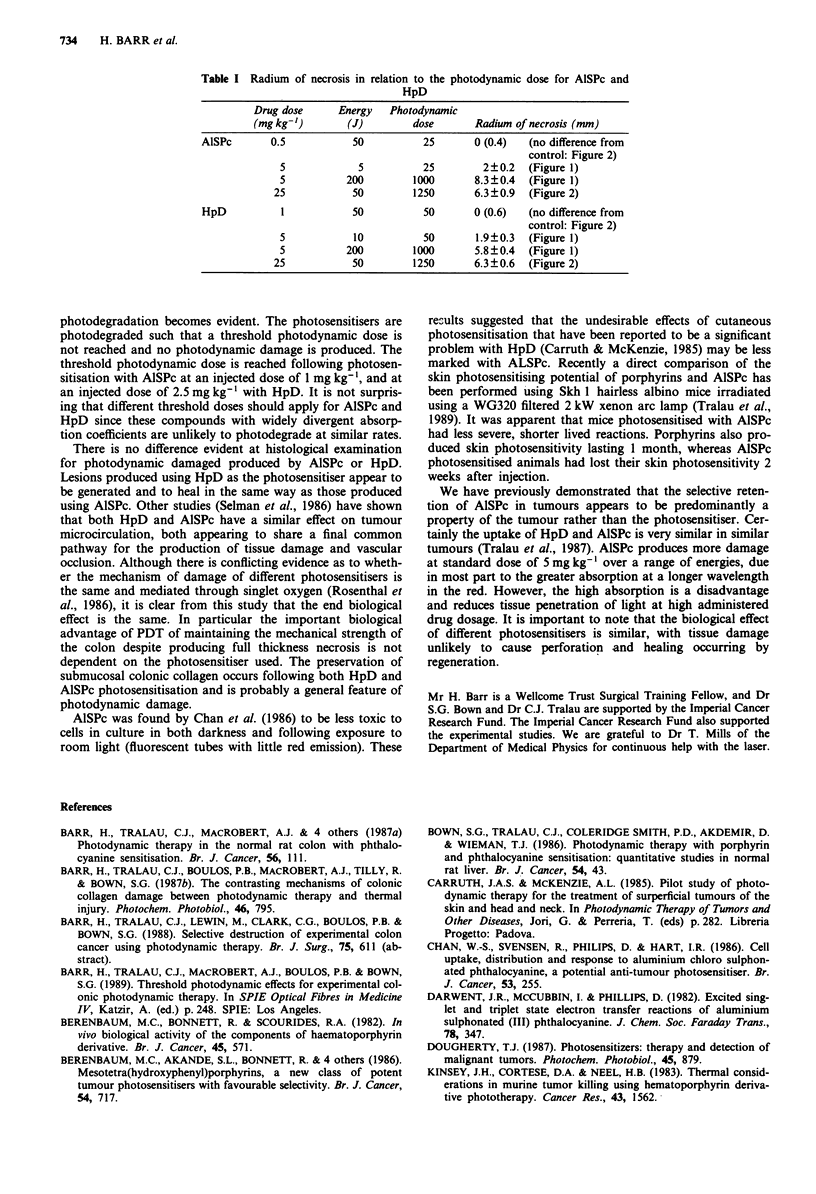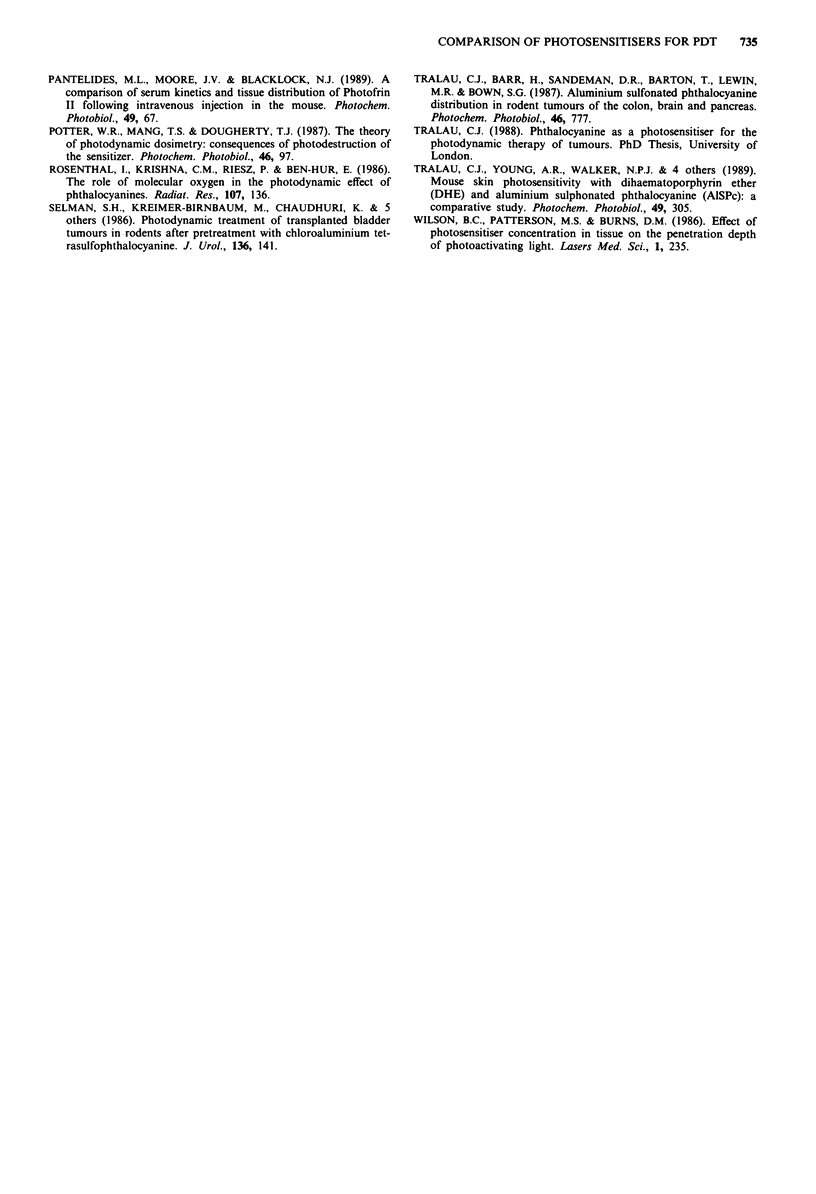# The significance of the nature of the photosensitizer for photodynamic therapy: quantitative and biological studies in the colon.

**DOI:** 10.1038/bjc.1990.368

**Published:** 1990-11

**Authors:** H. Barr, A. J. MacRobert, C. J. Tralau, P. B. Boulos, S. G. Bown

**Affiliations:** Department of Surgery, Rayne Institute, University College London, UK.

## Abstract

**Images:**


Br. J. Cancer (1990), 62, 730-735

© Macmillan Press Ltd., 1990

### The significance of the nature of the photosensitiser for photodynamic therapy: quantitative and biological studies in the colon

H. Barr, A.J. MacRobert, C.J. Tralau, P.B. Boulos & S.G. Bown

*National Medical Laser Centre, Department of Surgery, The Rayne Institute, University College London, 5 University Street, London WC1E 6JJ, UK.*

**Summary** Photodynamic therapy (PDT) depends on the interaction of light with an administered photosensitiser to produce a local cytotoxic effect. The most widely used photosensitiser is haematoporphyrin derivative (HpD), but newer photosensitisers such as aluminium sulphonated phthalocyanine (AlSPc) are promising. HpD and AlSPc have been compared as photosensitisers for colonic PDT in the rat. Quantitative analysis showed that following injection of a standard photosensitiser dose, AlSPc produced more damage than HpD with increasing energy (fluence). Alteration of the injected dose of photosensitiser did not produce a clear difference. There was a loss of reciprocity for photosensitiser/light combinations at low injected dose (0.5 mg $kg^{-1}$), both HpD and AlSPc producing no damage. Similarly at high photosensitiser dosage (25 mg $kg^{-1}$) there was no quantitative difference between AlSPc and HpD. Photosensitiser photodegradation at low photosensitiser doses, and light attenuation by high tissue concentrations of AlSPc account for these findings. PDT with either agent produced the same histological damage and full thickness necrosis produced no mechanical weakening of the colon measured by the bursting pressure. The submucosal collagen was preserved and healing was by regeneration.

The ideal photosensitiser for photodynamic therapy (PDT) has yet to be identified. Two groups of agents that are being extensively investigated are the phthalocyanines and the porphyrins. There are distinct chemical and photochemical differences between these agents. Haematoporphyrin derivative (HpD) is at present the most widely studied photosensitiser, and it is against this compound that other photosensitisers have to be compared. The major theoretical objections to HpD are that it is a variable mixture of porphyrins with only a small absorption peak in the red at 630 nm (Berenbaum *et al.*, 1982). We have compared this agent with aluminium sulphonated phthalocyanine (AlSPc). This was not a pure compound, but a mixture with varying degrees of sulphonation, although it has a large absorption peak at 675 nm (Bown *et al.*, 1986). For clinical PDT it is generally necessary to use a wavelength at the red end of the spectrum in order to get adequate tissue penetration of light. There are a few studies that directly compare the effectiveness of different photosensitisers (Berenbaum *et al.*, 1986), but no quantitative comparison of prophyrins and phthalocyanines.

These experiments compared the efficacy of both photosensitisers for colonic PDT for a range of light fluences and photosensitiser doses. We have previously reported in detail the effect of PDT on the normal rodent colon with AlSPc (Barr *et al.*, 1987*a,b*). These studies demonstrated that full thickness necrosis produced by photodynamic therapy following phthalocyanine photosensitisation did not reduce the mechanical strength of the colonic wall and healing was predominantly by regeneration. These findings offer very important biological advantages for the endoscopic treatment of gastrointestinal cancers with photodynamic therapy. Some small tumours may be destroyed without risking perforation and there is less chance of producing a stricture. It is important to determine whether these photodynamic effects are independent of the nature of the photosensitiser.

### Materials and methods

### *Photosensitisers*

Aluminium sulphonated phthalocyanine was obtained from Ciba-Geigy, and was dissolved in normal saline prior to injection. It contained an average of three sulphonic groups per molecule (Darwent *et al.*, 1982). The solution was kept in the dark but no other special precautions were taken.

Haematoporphyrin derivative was supplied by Paisley Biochemicals Ltd (Glasgow) and synthesised to yield approximately 60% of photosensitising esters. This solution was kept in the dark below O°C.

### *Photodynamic therapy*

Male Wistar (180–250 g) rats were used to compare HpD and AlSPc for colonic photodynamic therapy. All procedures were performed under general anaesthesia from intramuscular Hypnorm (fentanyl and fluanisone). The animals were injected intravenously (via the tail vein) with HpD or AlSPc under exactly the same conditions. A completely fresh sample of HpD was used each time. It is clear that prolonged storage of HpD and in particular refreezing of the sample can have quite profound effects on the photochemistry (Dougherty, 1987).

One hour following intravenous injection, a laparotomy was performed and the mobile portion of the colon was exteriorised onto the anterior abdominal wall. Initial experiments were also performed at 3, 48 and 168 h after photosensitisation with both photosensitisers. No difference was apparent in the nature or quantitative pattern of the damage. The largest lesions occured when phototherapy was 1 h after photosensitisation. Overall the largest areas of damage after photosensitisation with AlSPc occur following the profound vascular effects produced when phototherapy immediately follows photosensitisation (Barr *et al.*, 1987*a*). However, this effect was not so evident and less predictable following HpD photosensitisation (Bown *et al.*, 1986). Thus all quantitative studies were performed 1 h after photosensitisation.

A continuous-wave argon pumped dye laser (Cooper Laseronics, CA, USA) with the output coupled to a 0.2 mm quartz laser fibre was set to deliver 100 mW. The laser was tuned to 630 nm for treatment of animals photosensitised with HpD, and 675 nm for those treated with AlSPc. A portion of colon on the right side had any faecal matter gently milked away. The laser fibre was inserted into the lumen of the bowel by puncturing the colonic wall. It was threaded along the colon to a convenient point and held in a clamp just touching the colonic wall. The laser was switched on for the time planned for the exposure. The colon was kept moist with occasional irrigation with 0.9% saline. The laser

Correspondence: H. Barr.
Received 27 October 1989; and in revised form 10 May 1990.

fibre was removed and the entry site marked with a silk suture. The distance from this point to the treated area was measured with a micrometer. Control experiments were performed on unsensitised animals at both wavelengths with this laser power. Following exposure at 50 and 200 J (100 mW for 500 and 2,000 s), small areas of thermal damage associated with the tip of the laser fibre could be identified 72 h after treatment. There was no difference between the radius of necrosis associated with thermal damage at the different wavelengths. Those control data are shown in the figures. At lower energies thermal damage could only occasionally be identified at histological examination and was never seen macroscopically. Animals were treated under the following circumstances.

*Variable delivered energy*

The animals were sensitised with 5 mg $kg^{-1}$ AlSPc or HpD and treated 1 h after injection. The laser was set to deliver 100 mW for between 10 and 2,000 s altering the delivered energy from 1 to 200 J. The animals were allowed to recover and killed 72 h after treatment, and the colon was removed. The PDT lesions were sharply demarcated oval areas. The radius of necrosis was measured under the operating microscope (Wild M650), by measuring the two major radii at right angles to each other and taking the mean (Barr *et al.*, 1987*a*). All lesions were fixed in 10% formalin and prepared for histological examination.

*Variable photosensitiser dose*

Animals were sensitised with 0.5, 1, 2.5, 5, 10 and 25 mg $kg^{-1}$ of HpD or AlSPc. They were all treated 1 h after intravenous injection. The concentration of the photosensitiser was adjusted so that the volume of fluid injected was 0.75–1 ml. Phototherapy was delivered at the appropriate wavelength. The laser was set to deliver 100 mW for 500 s (50 J). The animals were allowed to recover and then killed 72 h after treatment, at which time there was a sharply demarcated lesion that could be measured as above.

*Temperature measurement during PDT*

Apart from local thermal damage associated with the tip of the laser fibre, any general rise in tissue temperature associated with laser irradiation may cause hyperthermic tissue destruction. It was important to know that there was no temperature rise in the colon apart from that at the small area of thermal injury associated with the laser fibre tip already identified. The temperature in the colonic wall was measured using an array of six copper/constantan microthermocouples inserted under the serosa of the colon, and connected to a seven-channel temperature logger. Using the operating microscope a small intravenous cannula was inserted under the serosa, the microthermocouple array was inserted through this and the cannula withdrawn. The laser fibre was placed as for PDT on the bowel mucosa as near as possible to the thermocouples. The temperature was measured at 630 nm and 675 nm with the laser set to deliver 100 mW for 1,000 s. The animal's core temperature was measured by a separate needle thermocouple inserted into the animal's thigh muscle.

*Measurement of the mechanical strength of the colon after PDT*

Photodynamic therapy of normal colon with AlSPc has been shown not to weaken the colonic wall. The method of gaseous distension to measure the bursting pressure has been previously described (Barr *et al.*, 1987*a*). Normal colon treated 1 h after photosensitisation with HpD or AlSPc was removed at 72 and 96 h (the time when histological full thickness necrosis is present) and the bursting pressure measured. The injected dose of photosensitiser was 5 mg $kg^{-1}$ and the laser was set to deliver 100 mW for 2,000 s (200 J). The method of treatment was as described above.

*Histology*

Animals were injected with 5 mg $kg^{-1}$ HpD and AlSPc and treated 1 or 48 h after with 100 mW for 500 s of light as described above. Animals were killed from 3 h to 3 weeks after treatment. The area of treatment was identified and fixed in 10% formalin and prepared for histological examination. They were stained with haematoxylin and eosin. In some sections an elastic-Van Gieson stain was used to demonstrate the submucosal collagen layer, which has been shown to be preserved following PDT with AlSPc (Barr *et al.*, 1987*b*).

### Results

*Variable light energy*

Figure 1 shows the results obtained by altering the delivered energy, for a standard injected dose of AlSPc and HpD. It is clear that HpD produces less damage than AlSPc and there are different energy thresholds to produce necrosis. Following photosensitisation with 5 mg $kg^{-1}$ HpD, 5 J of energy failed to produce a lesion, and the energy threshold was between 5 and 10 J. In contrast the energy threshold to produce necrosis with ALSPc was between 1 and 5 J at this injected dose.

*Variable photosensitiser dose*

Figure 2 shows the results of altering the administered dose of AlSPc and HpD. The radium of necrosis produced following photosensitisation with 0.5 mg $kg^{-1}$ AlSPc and 0.5 and 1 mg $kg^{-1}$ HpD was no greater than control. It is apparent that at low dose (0.5 mg $kg^{-1}$) and at high dose (25 mg $kg^{-1}$) there was no difference between the amount of necrosis produced by the different photosensitisers. However, at 1, 2.5, 5 and 10 mg $kg^{-1}$ HpD produces smaller lesions than AlSPc.

*Temperature measurement*

The maximum temperature measured from any thermocouple at 630 nm and 675 nm is shown in Figure 3. The temperature in the colonic wall remained at 31–34°C during irradiation. Since the colon was kept moist and exteriorised the temperature never reached the animal's core temperature and never reached a hyperthermic temperature (Kinsey *et al.*, 1983).

**Figure 1** Mean radius of PDT necrosis in normal colon as a function of the applied energy for a laser power of 100 mW, 1 h after injection of 5 mg $kg^{-1}$ of the photosensitiser. Each point represents the mean with the standard error of the mean from at least three separate animals. AlSPc (open square, continuous line), HpD (open circle, dotted line). Control lesion produced by 100 mW of laser light for 500 and 2,000 s (50 and 200 J) in unphotosensitised animals treated at 630 and 675 nm (filled triangle).

**Figure 2** Mean radius of PDT necrosis in normal colon as a function of the dose of administered photosensitiser. PDT performed 3 h after intravenous injection of the photosensitiser; light dose: 100 mW for 500 s (50 J). Each point represents the mean with standard error of the mean from at least three separate animals (five animals treated following photosensitisation with 25 mg kg$^{-1}$). AlSPc (open square, continuous line), HpD (open circle, broken line). Control (filled triangle): colon treated without photosensitisation with 100 mW for 500 s (50 J).

**Figure 3** Temperature recorded in the subserosal area of normal colon during PDT. Phototherapy performed 1 h after photosensitisation with HpD or AlSPc (5 mg kg$^{-1}$), with laser tuned to 630 nm (open triangle) or 675 nm (open circle) and a power of 100 mW. The animal's core temperature (filled square) was measured with a needle thermocouple in the thigh muscle. The temperatures recorded are the maximum obtained from any of the thermocouples (three recordings from separate animals).

*Mechanical strength of treated colon*

Figure 4 shows the bursting pressure of the colon after PDT with HpD and AlSPc photosensitisation. There is no reduction in the bursting pressure despite full thickness necrosis. Bursting did not occur through the PDT lesion.

*Histology*

Histological examination of the lesions produced by photodynamic therapy with HpD as the photosensitiser showed that there was no difference between the progression of damage and healing compared with AlSPc. The initial response of the tissue was evident in the blood vessels, with dilatation and haemorrhage in the submucosal vessels occurring within 3 h of light exposure. Figures 5 and 6 show colon 3 h after PDT with HpD and AlSPc respectively. The tissue appearances are exactly the same. All subsequent histological

**Figure 4** Mean bursting pressure of colon following PDT with HpD and AlSPc. Phototherapy performed 1 h after injection of 5 mg kg$^{-1}$ of photosensitiser, laser set to deliver 100 mW for 2,000 s (200 J). Each point represents the mean measurements from at least three animals (±s.e.m.). AlSPc (open square), HpD (open circle). The continuous line is the mean control bursting pressure from 11 separate animals.

**Figure 5** Section of normal colon 3 h after PDT with 100 mW for 500 s (50 J). Photosensitisation with 5 mg kg$^{-1}$ HpD, 1 h before light exposure. This section shows haemorrhage and oedema in the submucosal layer (H&E ×26).

sections showed no difference between the photosensitisers evident on microscopy and were the same as the findings presented previously (Barr et al., 1987a). Full thickness necrosis was evident at 48–96 h. Two weeks after phototherapy the colon had healed by regeneration.

Figure 7 shows that the submucosal collagen (stained with elastic-Van Gieson) was undamaged when HpD was the photosensitiser as was found with AlSPc (Barr et al., 1987a).

### Discussion

It was demonstrated by Bown et al. (1986) that photodynamic therapy using light energies from 1 to 200 J following photosensitisation with HpD produced less damage to the normal rat liver than following photosensitisation with AlSPc, after intravenous injection of a standard dose of 5 mg kg$^{-1}$. Our findings agree with this for a fixed dose of

**Figure 6** Section of normal colon 3 h after PDT with 100 mW for 500 s (50 J). Photosensitisation with 5 mg kg$^{-1}$ AlSPc, 1 h before light exposure. The findings are no different from those following photosensitisation with HpD (H&E ×26).

**Figure 7** Section to show submucosal colonic collagen 72 h after phototherapy (100 mW for 2,000 s, 200 J), photosensitisation with 5 mg kg$^{-1}$ HpD). A damaged blood vessel is seen in the centre with necrotic tissue around it. The collagen fibrils are seen to be intact and remain present after PDT (elastic-Van Geison, ×60).

photosensitiser (5 mg kg$^{-1}$) and a variable light dose. There is no report of a comparative study examining the two photosensitisers at a range of administered doses. It is clear that at 2.5, 5 and 10 mg kg$^{-1}$ the amount of damage is greater for AlSPc than HpD on a weight for weight basis. However, following injection of 0.5 mg kg$^{-1}$ AlSPc or HpD, there is no difference between the photosensitisers, since no photodynamic damage occurs (no different from control unsensitised animals). No photodynamic damage is evident also when the injected dose of HpD is 1 mg kg$^{-1}$. After injection of 25 mg kg$^{-1}$, there is also no difference between the photosensitisers; the amount of damage produced is exactly the same. The explanation is complex as there are two separate processes occurring, one at low dose and one at high dose.

It is clear that for AlSPc, at an injected dose of 25 mg kg$^{-1}$, the amount of damage is less than expected although, in view of the small number of animals used, some caution is required. In order to examine this effect, it is helpful to consider the 'photodynamic dose' (Wilson *et al.*, 1986). This can be defined as:

Photodynamic dose =
tissue concentration of photosensitiser × fluence

The tissue concentration of AlSPc at a given time after injection has an approximately linear relationship with the injected dose of photosensitiser (Tralau, 1988). If similar relationships are assumed for HpD, a more simple equation can be used.

Photodynamic dose = injected photosensitiser dose × energy

Using this approximation to construct Table I we have calculated the photodynamic dose to allow simple comparison of the different photosensitisers. At a photodynamic dose 1,000, the radius of necrosis with AlSPc (8.3 mm) is greater than that for HpD (5.8 mm). Consider the data in Table I to give a greater photodynamic dose of 1,250. Despite this increase, the amount of necrosis following AlSPc photosensitiation has fallen to 6.3 mm, whereas with HpD the amount of necrosis has increased to 6.3 mm. It is apparent, that on an increase in the photodynamic dose from 1,000 to 1,250, the amount of necrosis rises as expected following photosensitisation with HpD, but falls with AlSPc.

At high photosensitiser dose with a highly absorbing photosensitiser such as AlSPc the penetration of light in the tissues may be significantly reduced. Bown *et al.* (1986) noticed that for a fixed light dose the depth of necrosis in the liver first increased with increasing concentration of AlSPc then decreased. The fall off in effect was presumed to be due to absorption of light by large amounts of AlSPc in the liver reducing the optical penetration depth. They noted that at high doses the liver appeared almost black. There is also further evidence for this effect. Wilson *et al.* (1986) have performed an important series of experiments and theoretical analysis to discover the effect of photosensitiser concentration on the tissue penetration of red light with AlSPC. They state that the absorption coefficient of tissue with the addition of a photosensitiser can be given by:

Tissue absorption coefficient = inherent tissue absorption coefficient
+ absorption coefficient of photosensitiser

The absorption coefficient of the photosensitiser can be given by:

Absorption coefficient of photosensitiser =
concentration of the photosensitiser ×
specific absorbence of the photosensitiser at the wavelength used

Although there are no comparative data on HpD and AlSPc uptake in rat colon, studies of Photofrin II uptake in mouse colon have been performed (Pantelides *et al.*, 1989). When compared with AlSPc concentrations in rodent colon (Barr *et al.*, 1987*a*), there are similar levels of uptake. Therefore, there is unlikely to be a major difference due to the concentration in the colon. However, the extinction coefficient of HpD at 630 nm is $7 \times 10^{-4}$ mm$^{-1}$ ($\mu$g g$^{-1}$)$^{-1}$ and is 22 times lower than that of AlSPc at 675 nm (0.015 mm$^{-1}$ ($\mu$g g$^{-1}$)$^{-1}$) (Wilson *et al.*, 1986). Thus the tissue absorption coefficient of colon will be increased more when AlSPc rather than HpD is used as the photosensitiser. Therefore at high photosensitiser dose the light penetration could be reduced to such an extent that the energy threshold is not reached in the distal parts of the tissue. Therefore the amount of necrosis is lower than would be expected.

From Table I it is clear that at low dose there is a loss of reciprocity between the photosensitiser dose and the delivered energy. Following AlSPc photosensitisation a photodynamic dose of 25 produces no necrosis when the injected dose is 0.5 mg kg$^{-1}$ (energy 50 J), but 2 mm of necrosis if 5 mg kg$^{-1}$ (energy 5 J) is used. A photodynamic dose of 50 following photosensitisation with an injected dose of 1 mg kg$^{-1}$ HpD (energy 50 J) caused no damage, yet 2 mm of necrosis followed photosensitisation with 5 mg kg$^{-1}$ (energy 10 J). The simple definition of photodyamic dose given above breaks down at low photosensitiser doses. It has been shown that a threshold photosensitiser dose is required for necrosis to occur, whatever amount of energy is delivered (Barr *et al.*, 1989). It has also been demonstrated that photodegradation occurs with both HpD (Potter *et al.*, 1987) and AlSPc (Barr *et al.*, 1988). At low dose the occurrence of photosensitiser

**Table I** Radium of necrosis in relation to the photodynamic dose for AlSPc and HpD

| | *Drug dose ($mg\,kg^{-1}$)* | *Energy (J)* | *Photodynamic dose* | *Radium of necrosis (mm)* | |
|---|---|---|---|---|---|
| AlSPc | 0.5 | 50 | 25 | 0 (0.4) | (no difference from control: Figure 2) |
| | 5 | 5 | 25 | 2±0.2 | (Figure 1) |
| | 5 | 200 | 1000 | 8.3±0.4 | (Figure 1) |
| | 25 | 50 | 1250 | 6.3±0.9 | (Figure 2) |
| HpD | 1 | 50 | 50 | 0 (0.6) | (no difference from control: Figure 2) |
| | 5 | 10 | 50 | 1.9±0.3 | (Figure 1) |
| | 5 | 200 | 1000 | 5.8±0.4 | (Figure 1) |
| | 25 | 50 | 1250 | 6.3±0.6 | (Figure 2) |

photodegradation becomes evident. The photosensitisers are photodegraded such that a threshold photodynamic dose is not reached and no photodynamic damage is produced. The threshold photodynamic dose is reached following photosensitisation with AlSPc at an injected dose of 1 $mg\,kg^{-1}$, and at an injected dose of 2.5 $mg\,kg^{-1}$ with HpD. It is not surprising that different threshold doses should apply for AlSPc and HpD since these compounds with widely divergent absorption coefficients are unlikely to photodegrade at similar rates.

There is no difference evident at histological examination for photodynamic damaged produced by AlSPc or HpD. Lesions produced using HpD as the photosensitiser appear to be generated and to heal in the same way as those produced using AlSPc. Other studies (Selman *et al.*, 1986) have shown that both HpD and AlSPc have a similar effect on tumour microcirculation, both appearing to share a final common pathway for the production of tissue damage and vascular occlusion. Although there is conflicting evidence as to whether the mechanism of damage of different photosensitisers is the same and mediated through singlet oxygen (Rosenthal *et al.*, 1986), it is clear from this study that the end biological effect is the same. In particular the important biological advantage of PDT of maintaining the mechanical strength of the colon despite producing full thickness necrosis is not dependent on the photosensitiser used. The preservation of submucosal colonic collagen occurs following both HpD and AlSPc photosensitisation and is probably a general feature of photodynamic damage.

AlSPc was found by Chan *et al.* (1986) to be less toxic to cells in culture in both darkness and following exposure to room light (fluorescent tubes with little red emission). These results suggested that the undesirable effects of cutaneous photosensitisation that have been reported to be a significant problem with HpD (Carruth & McKenzie, 1985) may be less marked with ALSPc. Recently a direct comparison of the skin photosensitising potential of porphyrins and AlSPc has been performed using Skh 1 hairless albino mice irradiated using a WG320 filtered 2 kW xenon arc lamp (Tralau *et al.*, 1989). It was apparent that mice photosensitised with AlSPc had less severe, shorter lived reactions. Porphyrins also produced skin photosensitivity lasting 1 month, whereas AlSPc photosensitised animals had lost their skin photosensitivity 2 weeks after injection.

We have previously demonstrated that the selective retention of AlSPc in tumours appears to be predominantly a property of the tumour rather than the photosensitiser. Certainly the uptake of HpD and AlSPc is very similar in similar tumours (Tralau *et al.*, 1987). AlSPc produces more damage at standard dose of 5 $mg\,kg^{-1}$ over a range of energies, due in most part to the greater absorption at a longer wavelength in the red. However, the high absorption is a disadvantage and reduces tissue penetration of light at high administered drug dosage. It is important to note that the biological effect of different photosensitisers is similar, with tissue damage unlikely to cause perforation and healing occurring by regeneration.

Mr H. Barr is a Wellcome Trust Surgical Training Fellow, and Dr S.G. Bown and Dr C.J. Tralau are supported by the Imperial Cancer Research Fund. The Imperial Cancer Research Fund also supported the experimental studies. We are grateful to Dr T. Mills of the Department of Medical Physics for continuous help with the laser.

### References

BARR, H., TRALAU, C.J., MACROBERT, A.J. & 4 others (1987*a*) Photodynamic therapy in the normal rat colon with phthalocyanine sensitisation. *Br. J. Cancer*, **56**, 111.

BARR, H., TRALAU, C.J., BOULOS, P.B., MACROBERT, A.J., TILLY, R. & BOWN, S.G. (1987*b*). The contrasting mechanisms of colonic collagen damage between photodynamic therapy and thermal injury. *Photochem. Photobiol.*, **46**, 795.

BARR, H., TRALAU, C.J., LEWIN, M., CLARK, C.G., BOULOS, P.B. & BOWN, S.G. (1988). Selective destruction of experimental colon cancer using photodynamic therapy. *Br. J. Surg.*, **75**, 611 (abstract).

BARR, H., TRALAU, C.J., MACROBERT, A.J., BOULOS, P.B. & BOWN, S.G. (1989). Threshold photodynamic effects for experimental colonic photodynamic therapy. In *SPIE Optical Fibres in Medicine IV*, Katzir, A. (ed.) p. 248. SPIE: Los Angeles.

BERENBAUM, M.C., BONNETT, R. & SCOURIDES, R.A. (1982). *In vivo* biological activity of the components of haematoporphyrin derivative. *Br. J. Cancer*, **45**, 571.

BERENBAUM, M.C., AKANDE, S.L., BONNETT, R. & 4 others (1986). Mesotetra(hydroxyphenyl)porphyrins, a new class of potent tumour photosensitisers with favourable selectivity. *Br. J. Cancer*, **54**, 717.

BOWN, S.G., TRALAU, C.J., COLERIDGE SMITH, P.D., AKDEMIR, D. & WIEMAN, T.J. (1986). Photodynamic therapy with porphyrin and phthalocyanine sensitisation: quantitative studies in normal rat liver. *Br. J. Cancer*, **54**, 43.

CARRUTH, J.A.S. & MCKENZIE, A.L. (1985). Pilot study of photodynamic therapy for the treatment of surperficial tumours of the skin and head and neck. In *Photodynamic Therapy of Tumors and Other Diseases*, Jori, G. & Perreria, T. (eds) p. 282. Libreria Progetto: Padova.

CHAN, W.-S., SVENSEN, R., PHILIPS, D. & HART, I.R. (1986). Cell uptake, distribution and response to aluminium chloro sulphonated phthalocyanine, a potential anti-tumour photosensitiser. *Br. J. Cancer*, **53**, 255.

DARWENT, J.R., MCCUBBIN, I. & PHILLIPS, D. (1982). Excited singlet and triplet state electron transfer reactions of aluminium sulphonated (III) phthalocyanine. *J. Chem. Soc. Faraday Trans.*, **78**, 347.

DOUGHERTY, T.J. (1987). Photosensitizers: therapy and detection of malignant tumors. *Photochem. Photobiol.*, **45**, 879.

KINSEY, J.H., CORTESE, D.A. & NEEL, H.B. (1983). Thermal considerations in murine tumor killing using hematoporphyrin derivative phototherapy. *Cancer Res.*, **43**, 1562.

PANTELIDES, M.L., MOORE, J.V. & BLACKLOCK, N.J. (1989). A comparison of serum kinetics and tissue distribution of Photofrin II following intravenous injection in the mouse. *Photochem. Photobiol.*, **49**, 67.

POTTER, W.R., MANG, T.S. & DOUGHERTY, T.J. (1987). The theory of photodynamic dosimetry: consequences of photodestruction of the sensitizer. *Photochem. Photobiol.*, **46**, 97.

ROSENTHAL, I., KRISHNA, C.M., RIESZ, P. & BEN-HUR, E. (1986). The role of molecular oxygen in the photodynamic effect of phthalocyanines. *Radiat. Res.*, **107**, 136.

SELMAN, S.H., KREIMER-BIRNBAUM, M., CHAUDHURI, K. & 5 others (1986). Photodynamic treatment of transplanted bladder tumours in rodents after pretreatment with chloroaluminium tetrasulfophthalocyanine. *J. Urol.*, **136**, 141.

TRALAU, C.J., BARR, H., SANDEMAN, D.R., BARTON, T., LEWIN, M.R. & BOWN, S.G. (1987). Aluminium sulfonated phthalocyanine distribution in rodent tumours of the colon, brain and pancreas. *Photochem. Photobiol.*, **46**, 777.

TRALAU, C.J. (1988). Phthalocyanine as a photosensitiser for the photodynamic therapy of tumours. PhD Thesis, University of London.

TRALAU, C.J., YOUNG, A.R., WALKER, N.P.J. & 4 others (1989). Mouse skin photosensitivity with dihaematoporphyrin ether (DHE) and aluminium sulphonated phthalocyanine (AlSPc): a comparative study. *Photochem. Photobiol.*, **49**, 305.

WILSON, B.C., PATTERSON, M.S. & BURNS, D.M. (1986). Effect of photosensitiser concentration in tissue on the penetration depth of photoactivating light. *Lasers Med. Sci.*, **1**, 235.